# Editorial: Malnutrition in dysphagia: nutritional assessment and management in clinical practice

**DOI:** 10.3389/fnut.2024.1416797

**Published:** 2024-05-21

**Authors:** Akio Shimizu, Kohei Yamaguchi, Keisuke Maeda

**Affiliations:** ^1^Department of Food and Health Science, Faculty of Health and Human Development, University of Nagano, Nagano, Japan; ^2^Department of Palliative and Supportive Medicine, Graduate School of Medicine, Aichi Medical University, Nagakute, Aichi, Japan; ^3^Department of Geriatric Medicine, Hospital, National Center for Geriatrics and Gerontology, Obu, Aichi, Japan; ^4^Department of Dysphagia Rehabilitation, Tokyo Medical and Dental University, Tokyo, Japan; ^5^Nutrition Therapy Support Center, Aichi Medical University Hospital, Nagakute, Aichi, Japan

**Keywords:** deglutition disorders, malnutrition, oral health, sarcopenia, texture-modified diet

Malnutrition and dysphagia have been shown to be closely related (1), and the management and intervention of these conditions in clinical practice are crucial for improving prognosis and maximizing patient quality of life. Multiple factors, including age, oral status, and disease burden (1), contribute to these conditions and, in particular, attention should be paid to the diet provided to individuals with dysphagia, including texture-modified foods and thickened liquids. A previous study have shown that the consumption of texture-modified diets is cross-sectionally associated with malnutrition, sarcopenia, and decreased quality of life in individuals with dysphagia (1, 2). Because texture-modified diets require special cooking, it is believed that the nutrient density per unit weight is reduced such settings, compared to regular diets (1). It has also been suggested that texture-modified diets may negatively affect appetite, which underscores the need for improvement (1). Adequate nutritional management affects nutritional status and swallowing function, with evidence beyond just adults with dysphagia.

This Research Topic aims to collect studies about malnutrition and dysphagia, as well as their assessment and management in clinical practice. In this Research Topic, there are five papers covering the aspects mentioned above.

Trovato et al. reported that providing children with neurological diseases with enteral nutrition supplements, selected for nutritional composition, may reduce the psychological burden on the patient compared to surgical procedures. Therefore, comprehensive nutritional management should be implemented for individuals with dysphagia in the clinic.

As malnutrition is frequently observed in individuals with dysphagia, a uniform nutritional assessment method is important. Zhou et al. examined the effectiveness of various nutritional assessment methods in detecting malnutrition in individuals, including the Global Leadership Initiative on Malnutrition (GLIM) criteria, Subjective Global Assessment, Patient-Generated Subjective Global Assessment, and Prognostic Nutritional Index. In their study, the number of individuals who were identified as having malnutrition using the GLIM criteria was lower than the population identified using other criteria (Zhou et al.). Notably, the GLIM criteria assess both phenotypic and etiological malnutrition criteria (3), and the inclusion of many measures of the GLIM criteria may have influenced the observed malnutrition prevalence. Furthermore, Bian et al. conducted a systematic review and meta-analysis of malnutrition diagnosis accuracy using the GLIM criteria and found that the GLIM criteria had a significantly lower prevalence of malnutrition than other nutritional screening tools. It is noteworthy that the review by Bian et al. focused on the association between malnutrition, as assessed by the GLIM criteria, and mortality, indicating the validity of the GLIM criteria as diagnostic criteria for malnutrition. According to a statement by registered Japanese dietitians who specialize in the treatment of dysphagia, the GLIM criteria are the preferred method for assessing the nutritional status of individuals with dysphagia (1). Therefore, the use of the GLIM criteria as a nutritional status assessment tool in clinical practice is desirable.

Oral health management is an important aspect of dysphagia management, and determining the appropriate texture-modified foods requires an accurate assessment of masticatory function, in addition to swallowing function (4). Oral health status is widely recognized to be associated with eating behaviors, including appetite, chewing, and swallowing. In a case report, Ding et al. noted that, in a patient with dysphagia who was receiving treatment for carcinoma at the base of the tongue with radiotherapy, nutritional management combined with adequate parenteral nutrition and the provision of appropriate texture-modified diets improved the patient's nutritional status. In clinical practice, oral health management by dental professionals is essential for treatment strategies, including the nutritional management of oral cancer. Therefore, special attention should be paid to individuals with dysphagia and oral health management teams for these individuals should include dental professionals.

Finally, we emphasize that treating dysphagia requires cooperation between medical and nursing professionals, industry, government, and other stakeholders. Shen et al. conducted a review of the nutritional supply methods for individuals with dysphagia and reported a need for collaboration among scientists from different disciplines to develop specialized foods for individuals with dysphagia. Considering the daily lives of individuals with dysphagia, the ingestion of food at home is a major challenge, and access to convenient, nutritious, and appealing food is crucial for individuals with dysphagia. Medical and nursing professionals cannot accomplish product development alone.

In summary, individuals with dysphagia face multiple challenges in nutritional, oral, and social dimensions. The existing literature and evidence on this critically important topic remain insufficient, despite that many studies have been published on this topic. We believe that our report will help readers understand the complex issues and challenges associated with dysphagia.

## Author contributions

AS: Conceptualization, Project administration, Resources, Writing – original draft, Writing – review & editing. KY: Writing – review & editing. KM: Writing – review & editing.
